# The Effect of Treating Vitamin D Deficiency or Insufficiency on Serum Adiponectin, Leptin and Insulin Resistance of Type 2 Diabetes Mellitus Patients: A Pilot Study

**DOI:** 10.22037/ijpr.2020.112067.13512

**Published:** 2020

**Authors:** Afshin Gharekhani, Farzad Najafipour, Hananeh Baradaran, Parisa Tagharrobi, Haleh Rezaee

**Affiliations:** a *Department of Clinical Pharmacy, Faculty of Pharmacy, Tabriz University of Medical Sciences, Tabriz, Iran. *; b *Endocrine Research Center, Tabriz University of Medical Sciences, Tabriz, Iran. *; c *Department of Clinical Pharmacy, Faculty of Pharmacy, Tehran University of Medical Sciences, Tehran, Iran. *; d *Student’s Research Committee, Tabriz University of Medical Sciences, Tabriz, Iran.*

**Keywords:** Adiponectin, Insulin resistance, Leptin, Type 2 diabetes mellitus, Vitamin D

## Abstract

Vitamin D deficiency is considered as one of the most prevalent healthcare problems in the world. Vitamin D contributes to insulin synthesis and secretion. Deficiency of vitamin D leads to insulin resistance which is the major cause of type 2 diabetes mellitus. We aim to evaluate the effect of treating vitamin D deficiency or insufficiency on serum adiponectin, leptin, and leptin to adiponectin ratio (LAR) of type 2 diabetes mellitus patients. Forty patients with type 2 diabetes mellitus were included according to the inclusion criteria of the study. Fasting venous blood samples were obtained and evaluated before and after the treatment of vitamin D deficiency or insufficiency. Then, blood levels of leptin, adiponectin, and LAR (an indicator of insulin resistance) were measured. The results of study indicate a significant decline in circulating leptin and adiponectin after vitamin D treatment, but it doesn’t cause a noteworthy change in LAR. Furthermore, the study demonstrates that female gender, higher body mass index, and triglyceride levels increase LAR significantly. It was concluded that the treatment of vitamin D deficiency or insufficiency doesn’t change insulin resistance in diabetic patients. Moreover, we concluded that LAR is not a reliable method to compare insulin resistance between men and women due to sex-related differences in adipose tissue.

## Introduction

Vitamin D deficiency is one of the major healthcare problems in the world (1). Vitamin D has important roles in the biological and physiological activities of the body. In addition to its known functions in bone homeostasis and mineral metabolism, it performs the other non-skeletal activities, due to the presence of vitamin D receptors in various tissues of the body. The most important activities include regulation of immune responses, glucose homeostasis, regulation of cell division, and differentiation (2-4). Vitamin D deficiency is a risk factor that predisposes patients to type 2 diabetes mellitus (T2DM) since it impairs the production and secretion of insulin (3, 5, 6). In recent years, studies evaluated the association between vitamin D levels and the risk of insulin resistance in T2DM patients. Some studies have found a correlation between vitamin D deficiency and insulin resistance, but the rest of them didn’t report any significant correlation (7-13). 

There are different methods for assessment of insulin resistance, which in turn have their advantages and disadvantages. The gold standard method is Hyperinsulinemic Euglycemic Glucose Clamping (HEGC) method which was developed by DeFronzo *et al*. in 1979 (14). HEGC method was very difficult, costly, and time-consuming, so it wasn’t suitable for clinical settings. After that, the minimal model of a frequently sampled intravenous glucose tolerance test (FSIVGTT) was introduced as an alternative for HEGC. FSIVGTT was simpler than the HEGC method but it wasn’t suitable for large scales, too (15, 16). Consequently, researchers introduced new indirect methods, which were suitable for clinical settings and researches, including; Homeostatic Model Assessment of Insulin Resistance (HOMA-IR) and Quantitative Insulin Sensitivity Check Index (QUICKI) (17). Recently, several studies reported that HOMA-IR has some limitations in predicting insulin resistance in certain populations (18, 19), therefore two adiponectin-based methods were developed; the HOMA corrected by adiponectin (HOMA-AD) and leptin to Adiponectin ratio (LAR) (20, 21). Leptin and adiponectin are two peptide hormones derived from adipocytes. Leptin reflects the total body fat and it controls energy balance by increasing energy consumption and reducing food intake. Adiponectin is an anti-inflammatory and anti-atherogenic factor that has positive effects on the cardiovascular system and prevents insulin resistance. In obese patients, leptin serum levels and resistance to the effects of leptin increase. On the other hand, adiponectin concentration decreases in metabolic syndrome and T2DM. Leptin and adiponectin alterations in T2DM influences the adipose tissue function and increases insulin resistance. According to the results of studies, LAR has a similar sensitivity for insulin resistance measurement compared to the gold standard method (HEGC), moreover it predicts the cardiovascular risk better than the former indices (22-30). 

Most of the studies, which evaluated the insulin resistance and vitamin D deficiency correlation, used the HOMA-IR method for measuring insulin resistance (7-12). So, the role of treating vitamin D deficiency on insulin resistance hasn not yet been proven using sensitive indices such as the LAR method. Thus, we aim to evaluate the effect of vitamin D deficiency or insufficiency treatment on serum adiponectin, leptin, and LAR in T2DM patients.

## Experimental

*Material*

Leptin enzyme-linked immunosorbent assay (ELISA) kits were purchased from Mediagnost, Germany (Reference range of kit: 0.28-9.60 ng/mL for men and 2.10-28 ng/mL for women), also, Adiponectin ELISA kits were purchased from the same company (Reference range of kit:2.00-13.90 µg/mL for men and 4.00-19.40 µg/mL for women). Vitamin D3 ELISA kit was bought from IDS, England.

*Methods*

This before-after study was performed in the Endocrinology Clinic of the Tabriz University of Medical Sciences from April 7 to July 16 of 2016 and a total number of 40 patients were evaluated. We included 18-70 years old patients with 7 < HbA1C < 8% and vitamin D blood levels <30 ng/mL. the pregnant and breastfeeding women, the patients who had severe comorbidities (*e.g.* malignancies, renal failure, liver failure, decompensated heart failure, infections, neurologic and autoimmune disease) and the individuals, who had received vitamin D supplementation within 3 months, were excluded from the study. The patients were categorized into three groups based on the severity of vitamin D deficiency (Table 1) (31).

Ten milliliter of fasting venous blood samples were obtained and centrifuged for 10 min at 3000 rpm, within 30 min from sampling. The serum was separated and stored at -20 centigrade until the time of leptin, adiponectin, and vitamin D levels analysis. The patients received 50000 international units (IU) vitamin D3 orally per week for eight weeks and then, leptin, adiponectin, and vitamin D levels were measured again (31). Finally, the insulin resistance value was calculated via dividing the numerical value of serum leptin levels (expressed in ng/mL) by serum adiponectin levels (expressed in µg/mL) for each patient. The effect of vitamin D supplementation on LAR was reported as the primary outcome of the study and the effects of gender, age, body mass index (BMI), HbA1c, triglyceride (TG), fasting blood glucose (FBS) on LAR, were considered as secondary outcomes. 

All statistical analyses were carried out using SPSS version 16.0 (SPSS, Inc., Chicago, IL, USA). Categorical data were reported as number and percentage, whereas continuous data were expressed as mean ± standard deviation (SD). The distribution normality of continuous variables was investigated using quantile normal plot, normal probability plot, and Shapiro-Wilk test. The paired *t*-test or Wilcoxon matched-pairs signed-rank test was used to compare differences within the groups. In addition, considering wide variation in serum level of vitamin D and LAR during the study period, to estimate the time trend changes in intervention effect and including possible effects of covariates, we also fitted a population-averaged panel-data model by using generalized estimation equation (GEE) with autoregressive one correlation structure to account for within-group correlation structure of the individuals. *P*-value of less than 0.05 was considered as statistically significant.

*Ethical approval*

This clinical trial was approved by the ethics committee of Tabriz University of Medical Sciences (ethical code: TBZMED.REC.1394.942 and IRCT code: IRCT2015070623083N1). All procedures performed in the study involving human participants were in accordance with the ethical standards of Tabriz University of Medical Sciences and with the 1964 Helsinki declaration and its later amendments or comparable ethical standards.

*Informed consent*

Informed consent was obtained from all the individual participants included in the study.

## Results

**Figure 1:**
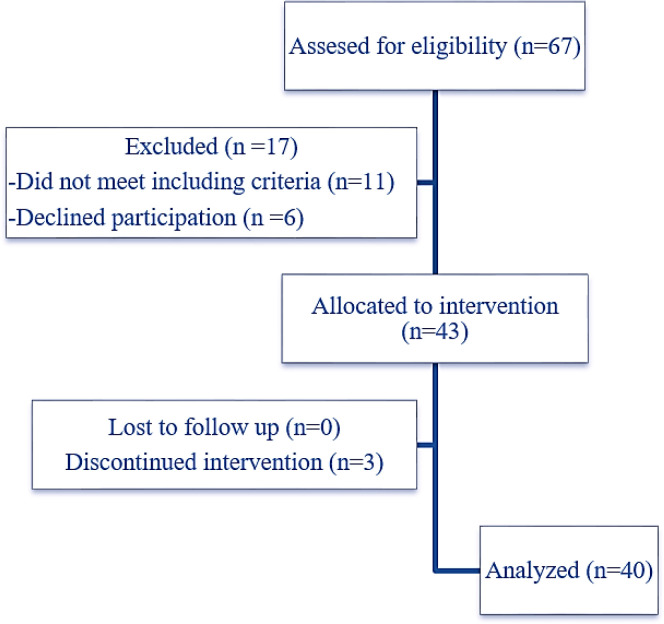
Flow diagram of assigned patients

Forty-three patients with T2DM were assigned in the study, three patients discontinued intervention and finally, forty patients (twenty males and twenty females) were included in the final analysis (Figure 1). The demographic information and paraclinical characteristics of the included patients are listed in Tables 2. In our study, most of the patients (47%) were overweighted, 15% were obese, 12% were very obese, 3% were morbidly obese and only 23% of patients had normal BMI. 

Forteen patients had vitamin D deficiency and twenty-six had vitamin D insufficiency. None of them were severely deficient. A total number of five patients had renal disease, three of them had a history of cardiovascular disease, three of them had eye problems and one patient had a thyroid disorder.

The results of the Wilcoxon test, which shows the comparison of leptin, adiponectin, vitamin D levels, and insulin resistance, before and after our intervention, are summarized in Table 3. 

After treatment, leptin, adiponectin, and vitamin D levels show significant differences compared to the baseline values (*P *< 0.05) but there isn’t any significant change in insulin resistance (*P* = 0.36). 

**Figure 2:**
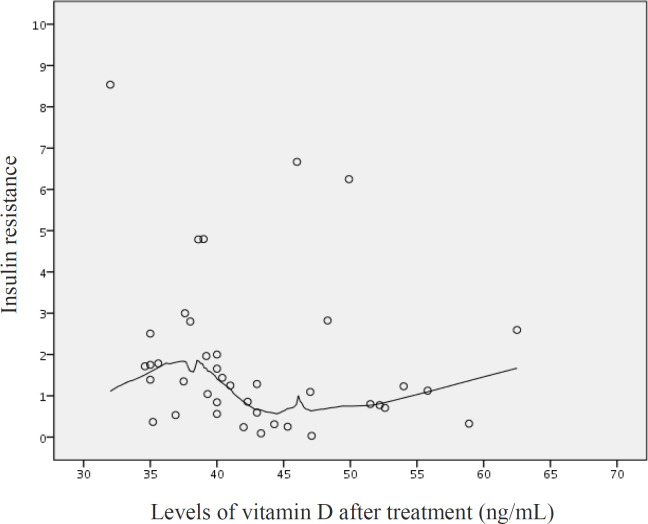
Distribution plot representing the correlation between vitamin D levels and insulin resistance, after treating vitamin D deficiency. Each ◦ represents each patient’s insulin resistance and vitamin D levels

The distribution plot of vitamin D levels and insulin resistance demonstrates a lack of correlation between two variables after the treatment (Figure 2). As it is obvious, the insulin resistance has been fixed between 0 to 3 in almost all patients, and there are a few participants with higher levels of insulin resistance. 

The GEE test results reveal that the leptin serum levels and insulin resistance are approximately 17 ng/mL and 2 units lower in men than women, respectively (Table 4). The age parameter illustrates 0.483 ng/mL increment in leptin serum levels per year, also, it reveals 0.454 ng/mL/year increment in adiponectin serum levels. In addition, the results demonstrate that a 1 point BMI increment raises leptin levels by 1.431 ng/mL and increases insulin resistance about 0.1 units. Furthermore, 1 ng/mL rise in vitamin D levels decreases leptin serum levels by 0.13 ng/mL, and reduces adiponectin serum levels by 0.163 ng/mL. Finally, as it is mentioned in Table 4, 1 mg/dL increment in TG serum levels decreases adiponectin levels by 0.51 ng/mL; however, it raises insulin resistance by 0.007 units.

## Discussion

 The principal pathophysiological feature of T2DM is the dysfunction of beta cells which is widely associated with obesity, metabolic syndrome, and insulin resistance. In addition, it seems that vitamin D deficiency contributes to insulin resistance and predisposes patients to T2DM (5). A study by Taheri *et al.* on 100 diabetic and 100 nondiabetic individuals, showed that vitamin D deficiency was more prevalent in diabetic patients (82.1% in diabetic and 75.6% in non-diabetic patients) (32). Furthermore, in another study, Bachali *et al. *revealed that levels of vitamin D in diabetic individuals were lower than non-diabetic individuals (2). Several studies evaluated the effects of vitamin D supplementation on glycemic indexes *e.g.* FBS, HbA1C, and insulin resistance, but they obtained contradicting results due to the differences in study design and interventions (7, 33-35). 

According to this controversy, we designed a study to evaluate the effect of vitamin D supplementation and also other suspected variables on leptin, adiponectin, and LAR. Our study indicated that treating vitamin D deficiency or insufficiency has a significant effect on serum levels of leptin and adiponectin, but does not have a meaningful effect on LAR. It may be because of the limited range of variation in vitamin D levels (30-65 ng/mL) according to the goal of therapy in this study. Thus, it seems possible to achieve a correlation between serum vitamin D and insulin resistance by a wider range of vitamin D serum levels. Our study confirms the results of a recent study which was conducted by Gulseth *et al.* and used the gold standard method of insulin resistance measurement (HEGC) for evaluating the consequences of vitamin D supplementation on insulin resistance. In this study, 62 diabetic patients were included and divided into control and intervention groups. The intervention group received a single oral dose of 400000 IU vitamin D3 then were evaluated after 6 months. The study found that this kind of vitamin D administration doesn’t improve insulin resistance (13). In the study of Patel *et al.* the participants (T2DM patients with vitamin D levels <25 ng/mL) were randomly divided into two groups. The first group received 400 IU/day and the second group received 1200 IU of vitamin D per day for four months. At the end of the fourth month, their insulin resistance was measured through the QUICKI method. This study failed to find any relation between receiving vitamin D supplements and controlling glucose levels of T2DM patients. Notably, the mean serum levels of vitamin D in the participants did not reach the normal levels (36). In another study, Heshmat *et al.* included 42 diabetic patients and found that there isn’t a correlation between vitamin D supplement injection and insulin resistance. This study focused on the calcium-dependent insulin secretion and concluded that concomitant use of vitamin D and calcium supplement could change the results (37). On the other hand, in the study of Baziar *et al.* prescribing vitamin D supplements for type 2 diabetic patients led to a significant reduction in insulin resistance level. In their study, 81 participants were divided into two groups. The first group received a vitamin D supplement (50000 IU/week for eight weeks) and the second group received a placebo. The insulin resistance of these participants was measured at the beginning of the study and at the end of receiving vitamin D supplement by the HOMA-IR method (38). According to the findings of the mentioned studies, we considered two hypotheses for these controversial correlations between serum vitamin D and insulin resistance; the first one is alterations in detecting insulin resistance by various methods with different sensitivities and the second one is altered goal of vitamin D treatment in different studies. 

Similar to the other studies, our results show that BMI has a meaningful effect on insulin resistance due to the higher leptin levels in obese patients, so weight loss is considered as an important strategy in increasing insulin sensitivity (39, 40). Moreover, our research confirms the results of the other studies, demonstrating that aging is a predictor factor for circulating leptin levels (40). 

In addition, the result of our study is similar to the findings of studies by Baba-Ahmadi *et al.* and Mantzoros *et al*. which showed an inverse relationship between levels of triglycerides and adiponectin (41, 42). Thus, due to the positive effects of adiponectin on insulin sensitivity and metabolic syndrome, it seems that TG lowering agents can be used as therapeutic options in improving the insulin sensitivity of diabetic patients.

Finally, our study illustrates higher leptin levels along with greater insulin resistance in the female gender. As we know, sex-related hormones and special fat distribution pattern in women (pear-shaped body) provide lower cardiometabolic risk compared to men, independent of higher body fat in women gender (43). Thus, it seems that the inability of the LAR method to detect the effect of fat distribution on insulin resistance (independent of total body fat and leptin) is an important disadvantage in predicting metabolic diseases. So, we suggest separating study groups by gender in studies that use the LAR method as an indicator of insulin resistance.

The significant reduction in adiponectin levels after vitamin D treatment was unexpected in our study. A few numbers of participants, laboratory errors, and the other unknown factors may have led to this issue. So, future studies with a large number of participants and more accurate laboratory tests are suggested. Other limitations in our study include the absence of a control group and lack of some other predictor information (*e.g.* decreased need for anti-diabetic agents), so it can be the subject of research in further studies.

**Table 1:** Classification of patients based on vitamin D levels

Vitamin D serum levels (ng/mL)	Definition
**20-29.99**	Insufficiency
**10-19.99**	Deficiency
**Below 10**	Severe Deficiency

**Table 2:** Demographic information and baseline paraclinical characteristics of patients

Parameter	Mean ± SD (range: minimum-maximum)
Age (year)	10.59 ± 55.37 (range: 29-70)
Height (cm)	9.94 ± 165.22 (range: 140-181)
Weight (kg)	14.32 ± 78.70 (range: 54-120)
BMI (kg/m^2^)	28.90 ± 5.22 (range: 20.76-46.87)
HDL (mg/dL)	49.55 ± 11.96
Cholesterol total (mg/dL)	158.45 ± 32.12
TG (mg/dL)	151.25 ± 79.06
HbA1c (%)	7.21 ± 0.63
FBS (mg/dL)	146.02 ± 43.04

**Table 3:** Leptin, adiponectin, vitamin D levels, and insulin resistance, before and after vitamin D supplementation (The Wilcoxon matched-pairs signed-rank test).

*P*-value	After vitamin D supplementation	Before vitamin D Supplementation	Variable
**<0.001**	15.46 ± 19.44	17.28 ± 22.42	Leptin (ng/mL)
**<0.001**	9.61 ± 14.34	10.48 ± 17.30	Adiponectin (g/mL)
**0.361**	1.90 ± 1.85	1.63 ± 1.72	Insulin Resistance (LAR)
**<0.001**	7.20±42.97	4.20±22.31	Vitamin D Levels (ng/mL)

**Table 4:** The results of the generalized estimation equation (GEE) test for evaluating the effects of predictor variables on leptin, adiponectin and insulin resistance

*P*-value	Estimated impact coefficient (β(	Estimated standard error	Wald statistic	Degree of freedom	**Variable**	Variable
**<0.001**	-16.92	3.56	22.55	1	Leptin	Gender
**0.208**	3.84	3.05	1.58	1	Adiponectin	
**<0.001**	-1.98	0.43	20.78	1	Insulin Resistance	
**<0.001**	0.48	0.11	19.18	1	Leptin	Age
**<0.001**	0.45	0.09	22.04	1	Adiponectin	
**0.449**	0.01	0.01	0.57	1	Insulin Resistance	
**<0.001**	1.43	0.39	13.03	1	Leptin	BMI
**0.354**	0.31	0.33	0.86	1	Adiponectin	
**0.002**	0.09	0.03	9.15	1	Insulin Resistance	
**0.138**	-0.07	0.04	2.20	1	Leptin	FBS
**0.242**	-0.05	0.04	1.37	1	Adiponectin	
**0.592**	0.01	0.01	0.28	1	Insulin Resistance	
**0.072**	0.03	0.02	3.23	1	Leptin	TG
**<0.001**	-0.05	0.01	12.41	1	Adiponectin	
**0.022**	0.01	.0032	5.285	1	Insulin Resistance	
**0.259**	1.47	1.31	1.27	1	Leptin	HbA1C
**0.336**	1.11	1.15	0.92	1	Adiponectin	
**0.785**	0.03	0.12	0.07	1	Insulin Resistance	
**<0.001**	-0.13	0.02	20.81	1	Leptin	Vitamin D
**<0.001**	-0.16	0.04	17.36	1	Adiponectin	
**0.091**	0.01	0.01	2.85	1	Insulin Resistance	

## Conclusion

The results showed that treating vitamin D deficiency decreases the circulating leptin and adiponectin but doesn’t have a significant effect on LAR. So, we concluded that vitamin D supplementation cannot alter insulin resistance directly, but it may have some beneficial consequences on diabetic patients due to its positive cardiovascular effects and leptin lowering properties. 
